# Editorial: HBV and lymphoma

**DOI:** 10.3389/fonc.2023.1236816

**Published:** 2023-07-13

**Authors:** Giuseppe Gentile, Luca Arcaini, Guido Antonelli, Maurizio Martelli

**Affiliations:** ^1^ Department of Translational and Precision Medicine, Sapienza University Rome, Rome, Italy; ^2^ Department of Molecular Medicine, University of Pavia and Division of Hematology, Fondazione Istituto di Ricovero e Cura a Carattere Scientifico (IRCCS) Policlinico San Matteo, Pavia, Italy; ^3^ Department of Molecular Medicine, Sapienza University, Rome, Italy

**Keywords:** hepatitis B virus, HBV, lymphoma, HBV lymphomagenesis, HBV reactivation, survival of patients with lymphoma and HBV, HBV-related cryoglobulinemia, prevention of HBV reactivation

Hepatitis B virus (HBV) infection is a global public health problem, being the highest prevalence observed in Asia, Africa and South America, and with over 257 million people worldwide infected and with estimated 887,000 deaths caused directly or indirectly by HBV every year. Even in the non-endemic countries, such as United States and Europe, approximately 1 and 13 million individuals, respectively, suffer from chronic HBV infection. Overall, approximately one-third of the world’s population have been infected and carry serological evidence of past or present HBV infection. In the recent years has been shown that viral hepatitis-related mortality exceeds the mortality attributed to tuberculosis, malaria and HIV (1).

HBV is a leading risk factor for the development of hepatocellular carcinoma, but epidemiological and clinical studies have also shown that HBV infection might increase the risk of extrahepatic malignancies such as non-Hodgkin lymphoma (NHL). In particular, individuals infected with HBV, in both Asia and North America, have a two-to threefold higher risk of developing NHL, mainly diffuse large B-cell lymphoma (2).

The etiopathological role of HBV in lymphomagenesis remains largely unknown. HBV can directly infect lymphocytes and integrate its nucleic acid sequences into the host’s genome, leading to the overexpression of oncogenes or the downregulation of tumor suppressor genes. Indeed, patients with NHL and HBV infection have the virus detected in their peripheral blood mononuclear cells (PBMC), in which HBV viral integration has also been observed. Furthermore, HBV viral replication and viral antigens may also stimulate the expression, production, and release of hematopoietic tumor growth factors, leading to clonal lymphocyte proliferation (3).

A relationship between chronic HBV infection and extrahepatic nonmalignant diseases is supported by increasing evidence, including, for example, mixed cryoglobulinemia vasculitis, panarteritis nodosa and glomerulopathies (4). The nucleotide analogs (NA) are administered for the treatment of the chronic HBV infections and have shown a similar efficacy in some manifestations of cryoglobulinemic vasculitis (CV).

The association between HBV infection and NHL has been demonstrated for HBsAg-positive patients, although patients with occult HBV infection have been shown to also be at high risk of NHL. Meta-analyses confirmed this association, regardless of the region and endemicity of HBV (2); even if, only a few studies have analyzed the characteristics of HBV-associated NHL in low-endemicity regions. Patients with lymphoma and current (positive hepatitis B surface antigen (HBsAg)) or past (positive hepatitis B core antibody (HBcAb) and negative HBsAg) HBV infection who receive chemotherapy, immunosuppressive therapies and steroids may develop a HBV reactivation (HBVr), potentially leading to interruption of chemotherapy and then adding significant morbidity(e.g. hepatitis flare, hepatic decompensation, and hepatic failure) and mortality (5). The highest rates of HBVr are typically observed during immunochemotherapy with the anti-CD20 monoclonal antibody, rituximab, especially when it is combined with cyclophosphamide, doxorubicin, vincristine, and prednisone chemotherapy (5). All patients with lymphoma and current or past HBV infection who receive chemo-immunotherapy, should receive prophylaxis for HBVr (5).

This Research Topic contains both reviews and original articles that deal with a range of topics on HBV and Lymphoma. In their study Svicher et al. reviewed data on HBV lymphotropism and its potential impact on lymphomagenesis. Epidemiological studies on the occurrence of lymphoma and HBV infection have been updated reinforcing the epidemiological strength of the association between HBV and lymphoma. Studies on the presence of HBV DNA in PBMC have been summarized and its integration in the exonic regions of some genes is considered a possible source of carcinogenesis. In addition, have been reported the results of *in vitro* studies of HBV infection of hematopoietic stem cells and lymphoid cells suggesting possible patterns of persistent infection and viral reactivation. Finally, some considerations on possible implications between HBV infection of blood cells and the possibility of new targets for eradication treatment have been reported. For example, the ideal goal of anti-HBV therapy is the achievement of an HBV functional cure, defined as a persistent HBsAg loss together with undetectable serum HBV-DNA off-therapy, reflecting the silencing of cccDNA transcriptional activity. It has been shown that HBV DNA integration in PBMC may represent a source of HBsAg even if cccDNA transcriptional activity has been blocked. Therefore, the evaluation of the efficacy of novel anti-HBV drugs should include the monitoring of HBV replication in PBMC. Wang et al. report on an elderly patient with primary biliary cirrhosis and with a new entity of NHL called fluid overload-associated large B-cell lymphoma (FOALBCL) being distinct from primary effusion lymphoma (PEL). Blood examinations excluded viral infections such as hepatitis B, C virus, Epstein-Bar virus and Kaposi sarcoma herpesvirus (KSHV)/human herpesvirus-8 (HHV-8) virus. Chest-computed tomography scan showed pleural effusion, early stage of cirrhosis, splenomegaly and abdominal-pelvic fluid accumulation. Cytological examination of the ascitic fluid showed large-size B-lymphocytes positive for CD20 and PAX5 and negative for HHV-8. After peritoneal drainage the clinical and hepatic conditions improved and the patient subsequently underwent successful chemotherapy. This clinical case underscores the relevance for both patients and clinicians in the identification and management of this new clinical entity updated in the 5^th^ edition of World Health Organization (WHO) Classification of Haematolymphoid Tumours (WHO-HAEM5). In addition, the prognosis of FOALBCL appears to be less aggressive, another reason to make the differential diagnosis from PEL. Mazzaro et al. reviewed the clinical manifestations and the effect of NA therapy on HBV-related CV. Among patients with HBV infection, CV is a rare event being observed in up to 5.5% of the patients with chronic hepatitis and the main clinical manifestations were mild-moderate, such as palpable leg purpura, asthenia and arthralgias observed in up to 90% of cases while severe manifestations such as leg ulcers, peripheral neuropathy, glomerulonephritis and NHL were less reported. The objective of the antiviral therapy is to obtain remission of HBV-related CV clinical manifestations by reducing HBV replication to undetectable HBV-DNA, therefore antiviral treatment in this setting is administered independently of severity of liver disease. The authors suggest that monotherapy with NA is the first option for HBV suppression and may give a good clinical response (reduction of cryoglobulins, normalization of rheumatoid factor and improvement of purpura on the leg, asthenia and arthralgia) in patients with mild-to moderate CV manifestations. Instead, in refractory patients the use of rituximab (anti-CD20 to eliminate cryoglobulin-producing B lymphocytes) alone or with plasma-exchange may be considered a therapeutic option in combination with NA therapy. Giordano et al. report a case-control study on 187 HBsAg-/HBcAb+ LNH patients undergoing R-CHOP-21. The patients were divided in 3 cohorts: in the first retrospective cohort, 96 patients were monitored for HBVr with monthly ALT and HBsAg test; in the second retrospective cohort, 60 patients received lamivudine (LAM) prophylaxis for 12 months; in the third prospective cohort, 31 patients received LAM prophylaxis for 24 months. In both the cohorts of patients who received 24-month LAM and 12-month LAM there were no episodes of immunochemotherapy disruption vs 7% observed in the pre-emptive cohort. HBVr was not observed in the 24-month LAM cohort compared to 10% observed in the 12-month LAM cohort and 12% diagnosed in pre-emptive group. The authors suggest that 24 months of LAM prophylaxis appeared to be effective and safe for hematological patients at high risk of HBVr. The lack of reliable biologic markers of immune recovery prevents the definition of the optimal duration of prophylaxis and the incidence of delayed HBV reactivation after the cessation of antiviral prophylaxis in lymphoma patients is unknown (5). Finally, Nuersulitan et al. showed in a retrospective study on 128 patients with DLBCL and chronic HBV infection that the 5-year progression-free survival and overall survival rates were 75.7% and 74.7%, respectively. Survival was comparable between patients with low and high HBV DNA levels. The authors underlie that patients with DLBCL and chronic HBV infection might obtain a good outcome if they receive concomitant cancer therapy and HBV prophylaxis. Recently, it has been reported in a large nationwide cohort from South Corea that untreated patients for chronic hepatitis B have had an increased risk of developing extrahepatic malignancies in particular NHL, being long term NA treatment associated with a lower risk of extrahepatic malignancies in this patient population *(* 6).

## Author contributions

GG drafted the manuscript, LA, GA and MM made substantial contributions to the work through discussion. All authors approved the submitted version.
